# CSF-Exosomal miRNAs and Delayed Cerebral Ischemia: Insights into Pathophysiology but No Definitive Biomarkers

**DOI:** 10.3390/biom15081161

**Published:** 2025-08-13

**Authors:** Chathathayil M. Shafeeque, Devin W. McBride, Yuanqing Yan, Hussein A. Zeineddine, John P. Hagen, H. Alex Choi, Jude P. Savarraj, Ari Dienel, Spiros L. Blackburn, Peeyush Kumar Thankamani

**Affiliations:** 1 Department of Neurosurgery, University of Texas Health Science Center, 6431 Fannin St. MSB 7.147, Houston, TX 77030, USA; 2 Feinberg School of Medicine, Northwestern University, Chicago, IL 60611, USA

**Keywords:** subarachnoid hemorrhage, CSF, exosome, miRNA, DCI and biomarker

## Abstract

Background: Aneurysmal subarachnoid hemorrhage (aSAH) is notoriously known for its high mortality and morbidity. Approximately one-third of the patients who survive aneurysm rupture are reported to develop delayed cerebral ischemia (DCI), which contributes to a poor clinical outcome. Currently, there are no biomarkers for identifying which aSAH patients are at risk of developing DCI. We aimed to determine the feasibility of cerebrospinal fluid (CSF) exosomal microRNAs (miRNAs) for predicting DCI post-aSAH. Methods: aSAH patients were prospectively enrolled, and CSF samples were collected at two time points (<24 h and 72 h post-aSAH) from individuals undergoing external ventricular drainage. Exosomal miRNAs were isolated from the CSF for analysis. In the initial group of patients (discovery cohort), an exploratory analysis was conducted using a CSF panel containing 84 miRNAs, assessed by quantitative real-time PCR (RT-qPCR). Based on this analysis, 27 miRNAs were selected for further evaluation in a second group of patients (validation cohort). Among these, 10 miRNAs had previously been reported in SAH-related CSF studies, supporting their relevance for continued investigation. Results: In this study, RT-qPCR analysis of 84 miRNAs in CSF samples from aSAH patients (n = 10 DCI, n = 16 no DCI) and non-aSAH controls (n = 5) identified 9 upregulated and 13 downregulated miRNAs in the DCI group, and 7 upregulated and 18 downregulated miRNAs in the no-DCI group, compared to the controls. When comparing DCI to no-DCI patients, 13 miRNAs were found to be upregulated in the DCI group. Additionally, seven miRNAs showed temporal upregulation in DCI patients between early (<24 h/T1) and later (72 h/T3) time points across both discovery and validation cohorts. However, no miRNAs were uniquely expressed in either DCI or no-DCI groups, limiting their potential as specific biomarkers for DCI. Conclusions: Despite analyses in both the discovery and validation phases, no miRNAs emerged as consistent and reliable biomarkers for distinguishing DCI from no-DCI patients. However, the identified miRNAs are involved in the key KEGG pathways that regulate vascular integrity, neuronal survival, and inflammatory processes central to DCI pathophysiology. These findings highlight the complexity of miRNA regulation following aSAH, as reflected by the variability in differentially expressed miRNAs between cohorts. This variability may be influenced by factors such as limited sample size, patient heterogeneity, individual biological differences, and experimental variability. Comprehensive profiling using larger, well-characterized cohorts, along with rigorous validation, is essential to determine the predictive value and mechanistic significance of candidate miRNAs in DCI.

## 1. Introduction

Aneurysmal subarachnoid hemorrhage (aSAH) is a global health burden that accounts for 5% of all strokes and is notoriously known for its high fatality and permanent disability rates [1,2]. While aSAH leads to pathophysiological events which contribute to early brain injury [3], it is delayed cerebral ischemia (DCI) that leads to significant morbidity and mortality [4]. DCI typically occurs between 3 and 14 days after the initial hemorrhage and is characterized by new focal neurological deficits or a decline in consciousness [5,6]. Its multifactorial pathophysiology includes cerebral vasospasm, microthrombosis, cortical spreading depolarizations, and impaired autoregulation, all contributing to secondary ischemic injury [7,8]. Approximately one third of aSAH patients who survive aneurysm rupture develop DCI [9]; thus, preventing or treating DCI after aSAH is of paramount significance as DCI leads to progressive brain damage, cognitive decline, and poor outcomes.

Currently, there is no reliable biomarker to accurately identify patients at risk of developing DCI following aSAH. Several studies have investigated serum biomarkers for inflammation [10,11] and platelet factors [12,13]. However, one limitation of serum biomarkers is that their levels can be affected by infection [14,15] and comorbidities [16]. Cerebrospinal fluid (CSF), which is in close contact with the extracellular space of the brain, has an intricate relationship with the brain, blood vessels, and blood products of aneurysmal rupture, meaning that it may be a better fluid to detect DCI biomarkers.

MicroRNAs (miRNAs) are small single-stranded molecules comprising 17–25 nucleotides, secreted by cells into the extravascular space and then circulated in the blood or CSF [17,18,19,20]. Physiologically, miRNAs promote degradation or interfere with the translation of mRNA and therefore regulate a broad range of cellular processes from normal development, homeostasis, and regeneration to apoptosis [21,22]. As of now, in humans, over 2000 miRNAs have been discovered and identified as specific for certain organs, tissue, or cells [22,23,24,25,26,27]. Brain-specific miRNAs that are enriched in neurites and synapses have also been identified [28,29,30,31,32,33,34].

Since exosomal miRNAs are released by cells and participate in signaling, rather than disposed of during cell death [35], exosomal miRNAs might be involved in the mechanisms responding to injuries. Our study aims to investigate exosomal miRNAs from CSF at two time points post-aSAH to evaluate their potential as predictive markers for the development of DCI. Therefore, we hypothesized that exosomal miRNAs in CSF could serve as potential biomarkers for identifying aSAH patients at risk of developing DCI.

## 2. Methods

### 2.1. Patient Enrollment

Under Institutional Review Board (IRB) of UTHealth approval (IRB Number: HSC-MS-22-0425, approved on 18 February 2023), patients admitted with aneurysmal subarachnoid hemorrhage (aSAH) were enrolled for the prospective collection of CSF and clinical data. Written informed consent was obtained from all participants or their legally authorized representatives prior to enrollment. The inclusion criteria were age ≥18 years and radiologically confirmed aneurysmal SAH. Patients with traumatic or non-aneurysmal SAH, malignancy, or systemic inflammatory disease were excluded. Per protocol, data were de-identified and stored in a prospective database. Age, sex, comorbidities, Glasgow Coma Scale (GCS), and Hunt–Hess score were collected and compared among the groups using Wilcoxon’s test or Fisher’s exact test for continuous and categorical variables, respectively. The aneurysmal source of bleeding was confirmed using computed tomography angiography (CTA). CSF samples were also collected from control patients undergoing lumbar puncture for neurological symptoms who had no history or radiographic evidence of any form of stroke. Controls were included if they were ≥18 years old and had no intracranial hemorrhage or CNS infection and were excluded if they had any prior cerebrovascular disease, CNS malignancy, or infection. A diagnosis of delayed cerebral ischemia (DCI), adjudicated prospectively by the NeuroICU faculty at a scheduled weekly meeting, was defined as a drop of 2 or more in GCS.

### 2.2. Sample Details

External ventricular drains (EVDs) were placed as standard care for SAH patients and used for serial CSF collection. CSF samples were collected at <24 (T1) and 72 h (T2) post-SAH, de-identified, and stored at −80 °C until analysis. All CSF samples underwent standard quality assessment prior to downstream processing, including visual inspection to ensure clarity and absence of visible blood contamination. Specimen collection and analysis occurred in two phases: discovery (26 aSAH patients, 5 controls) and validation (26 aSAH patients).

In the discovery cohort, CSF samples were obtained from 6 controls and 26 aSAH patients (10 DCI, 16 no DCI). Due to limited CSF availability in 8 no-DCI patients, samples from 2 patients were pooled, resulting in a total of 12 samples for the statistical analysis, comprising 8 individual and 4 pooled samples. Similarly, to address low CSF volume in some control samples, 1 pool consisting of 2 control samples was created, resulting in 4 individual controls and 1 pooled sample, for a total of 5 control samples. In the validation cohort, CSF was collected from 26 aSAH patients (12 no DCI, 14 DCI), all with sufficient volume for individual analysis.

The two-phase design allowed for an initial broad screening of miRNA expression changes in the discovery phase to generate hypotheses and identify candidate miRNAs potentially associated with aSAH and DCI. The validation phase then assessed the reproducibility and consistency of these candidates in an independent cohort. This approach balances sensitivity and specificity, helping to distinguish consistent miRNA alterations from random variation or cohort-specific effects, thereby laying the foundation for future biomarker studies.

### 2.3. Exosomal RNA Isolation and Quantification

The isolation and analysis of exosomes have been described previously [36]. Exosomes and RNA were isolated from 1 mL of CSF using the exoRNeasy Midi Kit (Qiagen, Hilden, Germany; Cat. No. 77144) and eluted to a final volume of 10 µL with nuclease-free water, following the manufacturer’s protocol. Reverse transcription of the isolated RNA was performed using the miRCURY LNA RT Kit (Qiagen; Cat. No. 339340). Real-time quantification assays were performed using the miRCURY LNA miRNA SYBR green PCR Assay Kit (Qiagen; Cat. No. 339346), which was optimized with a 1:10 dilution of cDNA.

In the discovery phase, RT-qPCR was performed using the LightCycler 480 instrument (Roche, Basel, Switzerland) on a 384-well plate with a Qiagen CSF miRNA panel, which includes pre-defined assays for 84 miRNAs expressed in exosomes from CSF (Table S1). After data analysis, 27 miRNAs with *p* < 0.1 in class comparisons (DCI vs. no DCI and T1 vs. T2) were selected for further validation in a new validation cohort (Table S2). Of these, 10 miRNAs had previously been reported in SAH-related CSF studies [37,38,39]. In the validation phase, RT-qPCR was conducted using Quant Studio 3 (Applied Biosystems, Waltham, MA, USA) to assess the selected miRNAs. In the validation phase, RT-qPCR was carried out using the QuantStudio 3 system (Applied Biosystems) with ABI-compatible 96-well plates (EarthOx, Chiyoda, Tokyo, Cat. No. PCRPH96). For reverse transcription, we used the Qiagen miRCURY LNA RT Kit (Hilden, Germany; Cat. No. 339340), and for amplification, the miRCURY LNA SYBR Green PCR Kit (Hilden, Germany; Cat. No. 339346) was used, following the manufacturer’s instructions. Based on Qiagen’s recommendations, *hsa-miR-191-5p* and *hsa-miR-103a-3p* were used as the controls for data normalization.

### 2.4. Statistical Analysis

We conducted linear regression analysis using GraphPad Prism (v10.0.2) to examine the demographic characteristics of the samples. Prior to statistical analysis with BRB-Array Tools (v4.6.2) [40], miRNA Ct values were transformed and normalized to prepare the dataset. For transformation, we subtracted each miRNA’s Ct value from a custom constant value of 50. This constant was selected because it exceeded the Ct values of the non-template controls (NTCs) in both cohorts, ensuring that all transformed values were positive. This transformation was applied to approximate a normal distribution, enhancing data stability and compatibility with BRB-Array Tools. Following transformation, the data were normalized to the average geometric mean of the control miRNAs to account for technical variation. We then used BRB-Array Tools (developed by Dr. Richard Simon and the BRB-Array Tools Development Team) to assess statistical differences in miRNA expression across the various group comparisons. A *p*-value threshold of <0.10 (raw *p*-values) was applied in both the discovery and validation phases to allow for the inclusion of miRNAs with potential biological relevance, minimizing the risk of false negatives in this CSF-based exploratory study, while validation in an independent cohort served to support the robustness of the findings. Additionally, pathway enrichment analysis for DCI-associated miRNAs was performed using the online tool miRPath v4.0.3.

## 3. Results

### 3.1. Baseline Characteristics of the Samples

**Figure 1:**
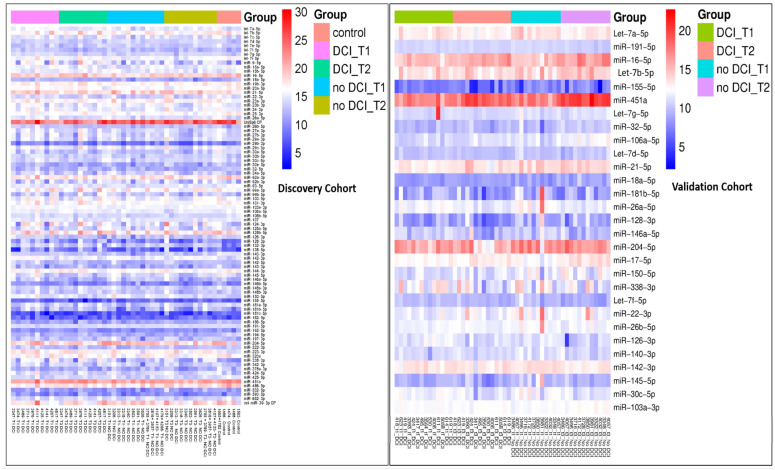
Heatmap showing clustering of miRNAs in the discovery and validation cohorts. The expression values within each group were represented, where red indicates higher expression and blue indicates lower expression. Overlap with previously reported SAH-associated miRNAs.

The demographics were compared among the groups. Overall, there were no significant differences in age, gender, or comorbidities between the SAH and control groups. However, the GCS scores differed significantly between the DCI and no-DCI groups within the validation cohort (*p* = 0.038). Additionally, the GCS scores also showed a significant difference between the discovery and validation aSAH groups (*p* = 0.023). In the subgroup analysis, hyperlipidemia (HLD) differed significantly between the discovery and validation cohorts within the no-DCI group (*p* = 0.044). Apart from these findings, no other significant differences were observed (Table 1). Hierarchical clustering of miRNAs in the discovery and validation cohorts is visualized using a heatmap (Figure 1).

Among the 27 miRNAs selected for validation (*p* < 0.1), several had been previously identified in CSF from SAH patients in independent studies, supporting the relevance of our candidate selection. Specifically, *let-7a-5p*, *let-7d-5p, hsa-miR-155-5p*, and *hsa*-*miR-451a* were reported by Pedrosa [38]; *hsa-miR-22-3p*, *hsa-miR-140-3p, hsa-miR-191-5p*, and *let-7b-5p* by Kikkawa [37]; and *hsa-miR-142-3p*, *hsa-miR-146a-5p*, *hsa-miR-150-5p*, *hsa-miR-451a*, and *let-7b-5p* by Wang [39]. Notably, *let-7b-5p* and *hsa-miR-451a* were consistently reported across multiple studies.

### 3.2. Differential miRNA Expression Pattern Between Control and aSAH

In the discovery phase of this study, we performed RT-qPCR using a Qiagen panel of 84 miRNAs on exosomes of CSF samples from 26 aSAH patients (n = 10 DCI, n = 16 no DCI) and 6 non-aSAH controls. Following statistical analysis, significant miRNAs with a *p*-value less than 0.10 (*p* < 0.10) were selected. We observed 9 upregulated and 13 downregulated miRNAs in the DCI group compared to the controls and 7 upregulated and 18 downregulated miRNAs in the no-DCI group compared to the controls.

**Figure 2:**
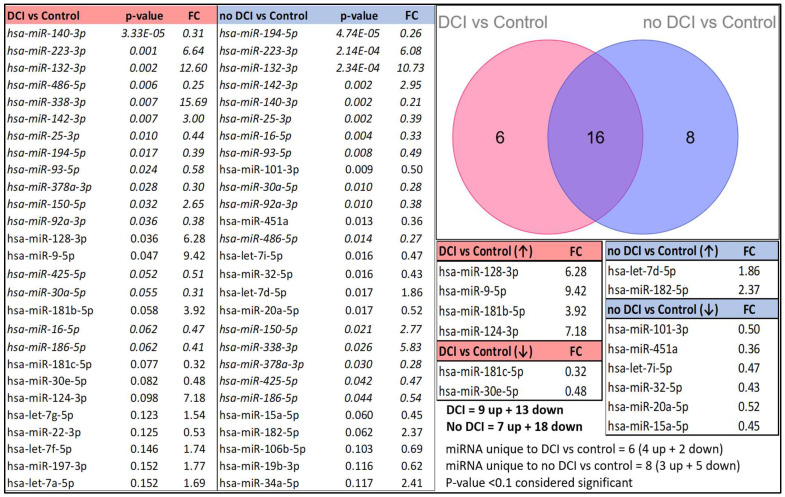
Differential miRNA expression patterns between the control and aSAH groups (DCI and no-DCI groups) are presented, sorted by *p*-values (*p* < 0.10). miRNAs that appear in both tables are italicized. A Venn diagram illustrates the number of unique and overlapping miRNAs in the DCI and no-DCI groups compared to the controls.

When comparing significant miRNAs between the controls vs. DCI/no-DCI groups, four miRNAs were uniquely upregulated and two were downregulated (out of six in DCI group vs. control), whereas two miRNAs were upregulated and six were downregulated (out of eight in no-DCI group vs. control) (Figure 2).

### 3.3. Differential miRNA Expression Pattern for DCI vs. No-DCI Groups

We aimed to identify a CSF exosome miRNA profile specific to the DCI cohort that could serve as a diagnostic or prognostic marker for individuals at risk of DCI. To achieve this, we conducted a comparative analysis between the DCI and no-DCI groups in both the discovery and validation cohorts. However, despite analyzing both cohorts, we were unable to confirm any miRNAs as reliable predictors of DCI, even though *hsa-miR-128-3p* and *hsa-miR-106a-5p* were present in both cohorts but exhibited opposite patterns of regulation in the discovery and validation phases (Table 2).

### 3.4. Temporal Changes in CSF Exosome miRNA Expression in Cohorts with and Without DCI

Our next goal was to examine temporal changes in miRNA expression in the DCI of both the discovery and validation cohorts between 24 h (T1) and 72 h (T2). Our analysis revealed that 13 miRNAs were upregulated between T1 and T2 in the discovery cohorts and 7 in the validation cohorts. Four miRNAs, *let-7a-5p*, *hsa-miR-126-3p*, *hsa-miR-146a-5p,* and *hsa-miR-26a-5p*, were common in both.

**Figure 3:**
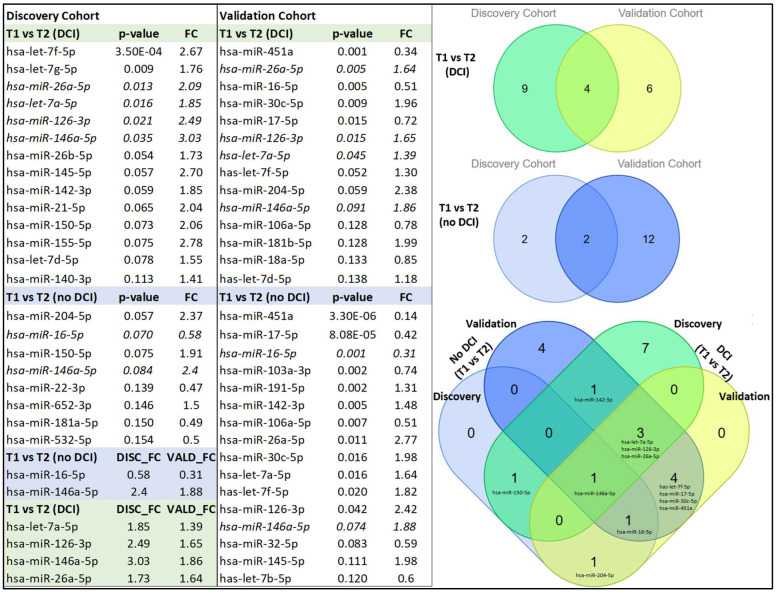
Temporal expression changes in exosomal miRNA in DCI and non-DCI cohorts (*p* < 0.10). miRNAs present in both tables are italicized. A Venn diagram depicts the number of overlapping miRNAs between T1 (<24 h) and T2 (72 h) within the DCI and non-DCI groups, with shades of blue and green indicating different categories. Additionally, a complex Venn diagram illustrates the overlap between the DCI and non-DCI groups, with miRNAs distinctly marked for clarity. FC: fold change from the (72 h) value.

Next, we compared miRNA expression at T1 and T2 in the no-DCI groups and identified three upregulated and one downregulated miRNAs in the discovery cohort. Upon validation, two miRNAs, *hsa-miR-16-5p* and *hsa-miR-146a-5p*, exhibited the same trend. Specifically, miRNA *hsa-miR-16-5p* was downregulated and miRNA *hsa-miR-146a-5p* was upregulated in both the discovery and validation cohorts (Figure 3).

**Figure 4:**
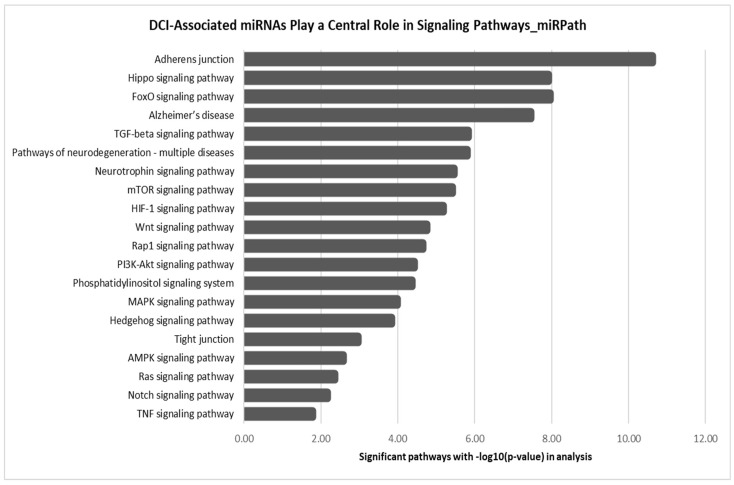
KEGG pathway enrichment analysis of DCI-associated miRNAs (*hsa-let-7a-5p*, *hsa-miR-126-3p*, *hsa-miR-146a-5p*, and *hsa-miR-26a-5p*), showing their involvement in key signaling pathways related to vascular integrity, neuroinflammation, and blood–brain barrier stability.

The temporal miRNAs identified in the DCI group—*let-7a-5p*, *hsa-miR-126-3p*, *hsa-miR-146a-5p*, and *hsa-miR-26a-5p*—were also present in the non-DCI group. Likewise, temporal miRNAs specific to the non-DCI group, such as *miR-16-5p* and *146a-5p*, exhibited similar regulatory patterns in DCI cases. As a result, their reliability as distinct biomarkers for DCI or non-DCI could not be validated. Although these miRNAs lack specificity as exclusive DCI biomarkers, their involvement in key signaling pathways (Figure 4) suggests potential roles in vascular integrity, neuroinflammation, and blood–brain barrier stability. To explore their functional relevance, we performed KEGG and Gene Ontology Biological Process (GOBP) enrichment analyses. While GOBP enrichment offered limited insight and is included as a Supplementary Figure (Figure S1), KEGG pathway analysis provided more informative direction, supporting the relevance of these miRNAs in broader mechanisms of neurovascular dysfunction and repair in DCI.

## 4. Discussion

Circulating miRNAs, known for their disease-specific alterations, have been investigated as diagnostic biomarkers for SAH and intracranial aneurysms, with their expression levels correlating with disease severity [41]. Among them, exosomal miRNAs have drawn particular interest for their potential to diagnose, predict, and monitor SAH prognosis [42]. However, their role in tracking DCI progression remains under-validated. In our study, we analyzed differentially regulated miRNAs in CSF samples from aSAH patients to identify molecular distinctions between the DCI and no-DCI groups. The observed differences in miRNA expression among the DCI, no-DCI, and non-aSAH control groups suggest that exosomal miRNA profiles are likely modulated by the aSAH condition, rather than implying a direct mechanistic role in DCI pathogenesis. miRNAs are associated with broader aSAH-related pathophysiological processes, such as inflammation, oxidative stress, and blood–brain barrier disruption [43,44,45], which are expected to be common in the DCI and no-DCI patient groups. There are some shared miRNAs between the DCI and no-DCI groups that underscore the need for further validation studies to refine the specificity of these candidates.

Our study aimed to identify a specific miRNA profile in CSF exosomes that could differentiate DCI from no-DCI cohorts, with the potential to serve as diagnostic or prognostic markers for managing individuals at risk of DCI. The contrasting expression patterns of *hsa-miR-128-3p* and *hsa-miR-106a-5p* with apparent upregulation in DCI patients in the discovery cohort but downregulation in the validation cohort warrant further investigation to clarify their roles in aSAH. For instance, *hsa-miR-128-3p* has been implicated in neuronal function and inflammatory responses [46,47], while *hsa-miR-106a-5p* is known to be involved in oxidative stress modulation and brain injury repair after ICH, particularly by influencing the activation of the Nrf2/ARE pathway [48]. The opposing expression patterns observed (Table 1) suggest that these miRNAs may have context-dependent roles, which may not be directly linked to DCI pathogenesis. Alternatively, their differential expression could reflect physiological variations rather than a specific mechanistic role in DCI. Further studies are needed to determine whether these miRNAs contribute to DCI development or are merely indicative of broader neurovascular changes.

The consistent upregulation of *let-7a-5p*, *hsa-miR-126-3p*, *hsa-miR-146a-5p*, and *hsa-miR-26a-5p* at critical time points (<24 h and 72 h) in both discovery and validation cohorts of DCI, as well as in the validation cohort of no-DCI patients (Figure 3), suggests their potential involvement in the molecular response to aSAH.

Among those potentially contributing to pathological processes, *let-7a-5p* has been implicated in inflammatory and immune responses and previously detected in plasma following aSAH [39]. Similarly, *hsa-miR-146a-5p*, a known regulator of inflammatory signaling cascades [49,50], may contribute to sustained neuroinflammation, which is a hallmark of DCI. Its elevated expression in DCI cohorts could reflect the amplification of inflammatory injury. *hsa-miR-26a-5p*, associated with cellular stress, apoptosis, and inflammation [51,52], may also reflect broader cell injury responses and ongoing pathological stress in the brain after SAH.

In contrast, *hsa-miR-126-3p*, which is strongly associated with endothelial function and vascular integrity [53], may represent a protective or compensatory response to vascular injury. Its upregulation may indicate attempts to preserve cerebrovascular stability in the face of early ischemic or inflammatory insults [54]. The presence of upregulated *hsa-miR-126-3p* and other miRNAs in the no-DCI group further supports the notion of shared, early adaptive mechanisms following aSAH, irrespective of subsequent DCI development.

The analysis of temporal changes in miRNA expression in the no-DCI group exhibited consistent expression patterns with *hsa-miR-16-5p* and *hsa-miR-146a-5p*, with the former being downregulated and the later one upregulated over time, possibly suggestive of their involvement in post-aSAH responses not leading to DCI. The downregulation of *hsa-miR-16-5p*, a known regulator of cellular stress, apoptosis, and inflammation [55], may indicate a protective or adaptive response aimed at mitigating pro-inflammatory signaling and fostering a more stable post-aSAH environment. Conversely, like that observed temporally in DCI, the upregulation of *hsa-miR-146a-5p* suggests that inflammatory processes remain active in SAH patients, contributing to cerebrovascular stability. However, the observation that *hsa-miR-16-5p* and *hsa-miR-146a-5p* exhibit similar temporal regulation in the DCI vs. no-DCI groups limits their specificity as biomarkers to identify aSAH patients at risk of DCI.

Despite the lack of differential expression, DCI miRNAs are involved in key KEGG pathways associated with cerebrovascular and neuroinflammatory processes. Pathways such as TGF-β, FoxO, mTOR, HIF-1, Wnt, and PI3K-Akt signaling are critical for maintaining vascular integrity, neuronal survival, and inflammation resolution, all central to DCI pathophysiology. Their involvement in pathways linked to adherens and tight junctions, Hippo, and MAPK signaling further suggests a role in blood–brain barrier stability and endothelial function, both of which are vital in post-SAH complications. Although their specificity as DCI biomarkers remains inconclusive, their regulation in neurodegeneration-related pathways, such as those linked to Alzheimer’s disease, highlights their broader relevance to neurovascular dysfunction. Rather than serving as exclusive biomarkers, these miRNAs may reflect overarching mechanisms of vascular stress, neuroinflammation, and repair responses, contributing to a deeper understanding of DCI pathogenesis. To refine their utility as biomarkers and therapeutic targets, future studies should investigate the mechanistic roles of these miRNAs in endothelial dysfunction, inflammation, and neuronal stress, exploring their differential regulation in DCI versus no-DCI groups.

## 5. Limitations

Our study highlights the inherent challenges in discovering robust biomarkers for predicting DCI, reflecting the complex and multifactorial nature of the condition. The variability in the number of differentially expressed miRNAs between discovery and validation cohort points to the complexity of miRNA regulation and the influence of various factors, including sample size, patient heterogeneity, individual patient variability, and the dynamic nature of miRNA regulation post-aSAH. Future studies with larger and more diverse patient populations, as well as more frequent sampling across multiple time points of blood and CSF, will be necessary to confirm these findings and fully elucidate the temporal dynamics of miRNA expression in DCI.

## 6. Conclusions

Our investigation identified a consistent upregulation of *hsa-let-7a-5p*, *hsa-miR-126-3p*, *hsa-miR-146a-5p*, and *hsa-miR-26a-5p* at critical time points (<24 h and 72 h) in DCI patients across both the discovery and validation cohorts, and notably also in the no-DCI validation group. These patterns suggest a shared early molecular response following aSAH. While no single miRNA reliably distinguished DCI from no-DCI patients, the repeated elevation of select miRNAs highlights their potential relevance in the post-SAH inflammatory or injury cascade. These findings support the idea that a combinatorial or pathway-based approach, possibly integrating multiple miRNA signatures and clinical variables, may be more effective in predicting DCI. Our results underscore the need for continued, large-scale profiling and validation to refine the predictive utility of miRNA biomarkers in aSAH.

## Figures and Tables

**Table 1:** Demographic and clinical characteristics of study participants across discovery and validation cohorts. The table summarizes the demographic and clinical variables of the participants in the discovery and validation cohorts, including age, gender, comorbidities such as hypertension (HTN), hyperlipidemia (HLD), and diabetes mellitus (DM), and Glasgow Coma Scale (GCS) scores.

Demographics	Discovery Cohort SAH: DCI vs. No DCI			Validation Cohort SAH: DCI vs. No DCI			Discovery SAH vs. Validation SAH			Discovery SAH_No DCI vs. Validation SAH_No DCI			Discovery SAH_DCI vs. Validation SAH_DCI		
	No DCI (N = 16)	DCI (N = 10)	*p*-Value	No DCI (N = 12)	DCI (N = 14)	*p*-Value	Discovery (N = 26)	Validation (N = 26)	*p*-Value	Discovery (N = 16)	Validation (N = 12)	*p*-Value	Discovery (N = 10)	Validation (N = 14)	*p*-Value
Age			0.786						0.229			0.237			0.584
Mean (SD)	51.6	50.6		58.3	53.3	0.433	51.2	55.6		51.6	58.3		50.6	53.3	
Gender			0.692			0.170									1.000
Male	5	4		1	5		9	6	0.541	5	1	0.196	4	5	
Female	11	6		11	9		17	20		11	11		6	9	
Race			0.538			0.836			0.276			0.568			0.948
American Indian or Alaska native	0	0		0	0		0	0		0	0		0	0	
Black or African-American	1	2		3	4		3	7		1	3		2	4	
White	15	8		8	9		23	17		15	8		8	9	
Asian	0	0		1	1		0	2		0	1		0	1	
Hypertension (HTN)			0.109			0.665			0.382			0.276			1.000
No	9	2		4	3		11	7		9	4		2	3	
Yes	7	8		8	11		15	19		7	8		8	11	
Hyperlipidemia (HLD)			1.000			0.090						*0.044*			1.000
No	14	9		6	12		23	18	0.173	14	6		9	12	
Yes	2	1		6	2		3	8		2	6		1	2	
Diabetes Mellitus (DM)			1.000			0.598			0.668			1.000			0.615
No	15	9		11	11		24	22		15	11		9	11	
Yes	1	1		1	3		2	4		1	1		1	3	
Tobacco Use			0.425			1.000			0.095			0.125			0.678
No	5	5		8	9		10	17		5	8		5	9	
Yes	11	5		4	5		16	9		11	4		5	5	
Alcohol Use			1.000			1.000			0.165			0.445			0.408
No	6	4		7	9		10	16		6	7		4	9	
Yes	10	6		5	5		16	10		10	5		6	5	
Aspirin			1.000			1.000			0.116			0.231			0.341
No	13	9		7	5		22	16		13	7		9	9	
Yes	3	1		9	5		4	10		3	5		1	5	
Plavix			1.000			0.462			1.000			0.429			1.000
No	16	10		11	14		26	25		16	11		10	14	
Yes	0	0		1	0		0	1		0	1		0	0	
GCS (3–15)			0.141			*0.038*			*0.023*			0.428			0.053
Mean (SD)	13.1	11.5		12	8.6		12.5	10.2		13.1	12		11.5	8.6	
Hunt Hess on arrival (1–5)			0.440			0.516			0.248			0.516			0.459
Mean (SD)	2.8	3		3	3.2		2.9	3.1		2.8	3		3	3.2	
Fischer Scale			0.385			0.462			0.186			0.429			0.417
1	0	0		1	0		0	1		0	1		0	0	
2	0	0		0	0		0	0		0	0		0	0	
3	16	9		11	14		25	25		16	11		9	14	
4	0	1		0	0		1	0		0	0		1	0	

**Table 2:** List of differential miRNA expression between DCI vs. no-DCI groups sorted based on *p*-values less than 0.10. miRNAs that appear in both tables are italicized. FC: fold change from the no-DCI value.

Discovery Cohort			Validation Cohort		
DCI vs. No-DCI	*p*-Value	FC	DCI vs. No-DCI	*p*-Value	FC
hsa-miR-194-5p	0.045	1.50	hsa-miR-21-5p	0.001	0.59
hsa-let-7i-5p	0.051	1.50	*hsa-miR-106a-5p*	*0.001*	*0.60*
*hsa-miR-128-3p*	*0.066*	*2.70*	hsa-miR-204-5p	0.007	0.41
hsa-miR-338-3p	0.078	2.69	hsa-miR-145-5p	0.016	0.54
hsa-miR-20a-5p	0.079	1.35	hsa-miR-22-3p	0.028	0.54
hsa-miR-138-5p	0.084	3.14	*hsa-miR-128-3p*	*0.030*	*0.48*
hsa-miR-18a-5p	0.088	1.49	hsa-miR-150-5p	0.032	0.60
hsa-miR-106b-5p	0.098	1.28	has-let7b-5p	0.033	0.64
hsa-miR-181b-5p	0.098	2.38	has-let7a-5p	0.040	0.76
*hsa-miR-106a-5p*	*0.100*	*1.30*	hsa-miR-451a	0.064	0.56
hsa-let-7d-5p	0.103	0.78	hsa-miR-126-3p	0.073	1.53
hsa-miR-9-5p	0.106	2.60	hsa-miR-26a-5p	0.098	0.69
hsa-miR-140-3p	0.113	1.48	hsa-miR-26b-5p	0.118	0.78

## Data Availability

The original contributions presented in this study are included in the article/Supplementary Materials. Further inquiries can be directed to the corresponding authors.

## Supplementary Materials

The following supporting information can be downloaded at: https://www.mdpi.com/article/10.3390/biom15081161/s1, Figure S1. Top-10 enriched Gene Ontology Biological Process (GOBP) terms for predicted targets of DCI-associated miRNAs. Table S1. List of 84 miRNAs included in the Qiagen. Table S2. Exosomal miRNAs selected for validation.
