# Supplementary figures and images for: CSF-Exosomal miRNAs and Delayed Cerebral Ischemia: Insights into Pathophysiology but No Definitive Biomarkers
Source: Biomolecules. 2025 Aug 13;15(8):1161. doi: 10.3390/biom15081161 (PMC12384643; doi:10.3390/biom15081161)

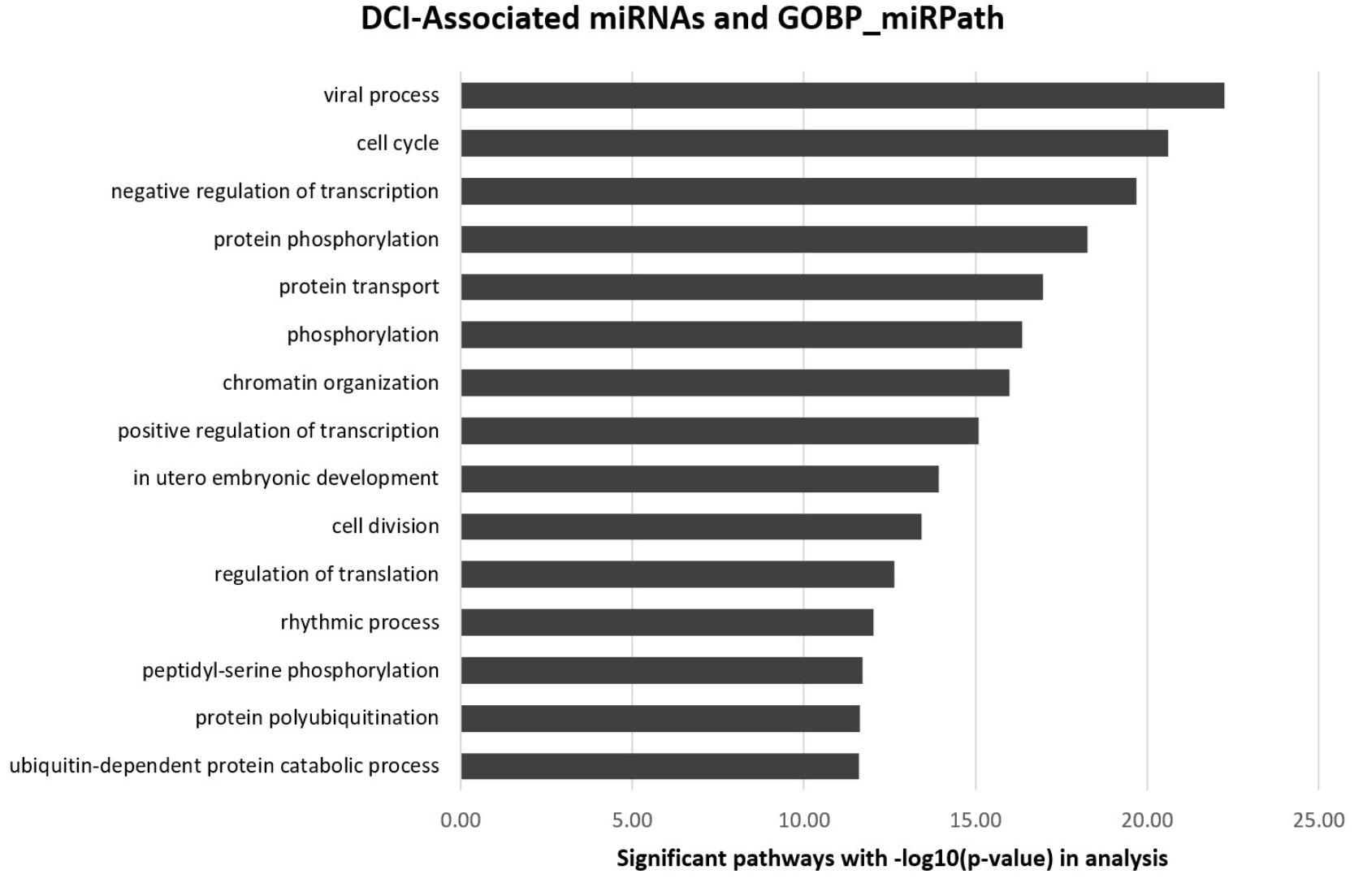

Supplement: Supplementary file 1 [file biomolecules-15-01161-s001.zip › Figure S1.jpeg]
